# Supplemental data for the paper “low-complexity detection of small frequency deviations by the generalized LMPU test”

**DOI:** 10.1016/j.dib.2020.106714

**Published:** 2021-01-08

**Authors:** Eyal Levy, Tirza Routtenberg

**Affiliations:** School of Electrical and Computer Engineering, Ben-Gurion University of the Negev, Beer-Sheva 84105, Israel

**Keywords:** Locally most powerful, Unbiased test, Nuisance parameters, Low-complexity methods, Frequency deviation

## Abstract

This document contains supplemental material for the paper [2]. The notations in this document are the same as in [2]. In particular, we first present here the proof of Theorem 1 in [2]. This theorem expresses the locally most powerful unbiased (LMPU) test, which is a general method for local detection, in the presence of known nuisance parameters. Second, we present here the Matlab code of the LMPU and the generalized LMPU for the special case of detection of a small deviation in the frequency of sinusoidal signals, which arises in various signal processing applications.

## Specifications Table

**Table:** 

Subject	Data Mining and Statistical Analysis
Specific subject area	Detection Theory
Type of data	Mathematical derivations, MATLAB code
How data were acquired	All source codes written in Matlab software.
Data format	Raw
Parameters for data collection	All the codes were implemented in MATLAB-R2019a on a system with Intel Core(TM) i7-10TH GEN CPU computer,2.80 GHz.
Description of data collection	The MATLAB code was configured as a function. The experiments were conducted by generating Monte-Carlo simulations of the model from (1) in [2], where the noise is generated by the Matlab function: wgn(N,’complex’), in which N is the number of measurements.
Data source location	All relevant data is contained in this paper
Data accessibility	With the article
Related research article	E. Levy and T. Routtenberg, “Low-complexity detection of small frequency deviations by the generalized LMPU test,” *Signal Processing*, https://doi.org/10.1016/j.sigpro.2020.107851.

## Value of the Data

• This document contains the proof of Theorem 1 in [2], which expresses the LMPU test that is a function of a known parameter vector, $\theta_{n}$. This is used in [2] to develop the GLMPU. This proof is important for the sake of completeness and since the LMPU it is less widely used than other detectors.
• This proof can be used in the future for developing new tests for various scenarios.
• The provided codes can be used for the detection of frequency deviations with low complexity.

## 1 Data Description

This article contains supplementary material for the paper in [2]. Specifically, it contains the proof of Theorem 1 in [2] and the code files in Matlab that were used to generate the graphs in [2]. Theorem 1 in [2] presents the locally most powerful unbiased (LMPU) test, which is a general test that maximizes the probability of detection under the α-size and unbiasedness constraints. This theorem presents the explicit test as a function of the likelihood function and as a function of the unknown nuisance parameters. The Matlab code contains the functions of the LMPU and generalized LMPU (GLMPU) tests that were used to generate Figs. 1–7 in [2], and is attached to this paper.

## 2 Experimental Design, Materials and Methods

 

## 3 Proof of Theorem 1 in [2]

A general non-random test, based on the observation vector, x, can be defined as

(3.1) $$\begin{array}{c} \Phi \left(x\right)\overset{▵}{=}\left\{ \begin{array}{cc} 1, & x\in S_{1}\\ 0, & x\notin S_{1} \end{array}\right., \end{array}$$

where $S_{1}\subset \Omega_{x}$ is the rejection region, which includes the values of x that lead to rejection of $H_{0}$ (acceptance of $H_{1}$).

In (6) in [2] we describe the general two-sided composite hypothesis testing problem, in which $\theta_{l}=\theta_{0}$ under the null hypothesis and $\theta_{l}\neq \theta_{0}$ under the alternative hypothesis, and $\theta_{n}$ is a nuisance parameter vector that appears under both hypotheses.

The power function of a general test (3.1), Φ, for this hypothesis testing is defined by (p. 69 in [1]):

(3.2) $$\beta_{\Phi }\left(\theta_{l},\theta_{n}\right)\overset{▵}{=}\int_{\Omega_{x}}\Phi \left(x\right)f\left(x;\theta_{l},\theta_{n}\right)dx,\;\;\;\theta_{l}\neq \theta_{0}.$$

The pdf of x under both hypotheses, $f(x;\theta_{l},\theta_{n}),$ is assumed to be a continuous and twice differentiable function w.r.t. the local parameter, $\theta_{l}\in R,$ for any unknown nuisance parameter vector, $\theta_{n}\in C^{K}$. Therefore, the power function $\beta_{\Phi }\left(\theta_{l},\theta_{n}\right)$ is also a continuous function w.r.t. the local parameter, $\theta_{l}\in R,$ especially at $\theta_{l}=\theta_{0}$.

A level-α, unbiased test, Φ, is said to be the LMPU test (p. 340 in [1]) if, for any other given level-α, unbiased test $\tilde{\Phi },$ there exists δ such that

(3.3) $$\beta_{\Phi }\left(\theta_{l},\theta_{n}\right)\geq \beta_{\tilde{\Phi }}\left(\theta_{l},\theta_{n}\right),\;\;\;\forall \theta_{l}\in \Omega_{\delta },$$

where $\Omega_{\delta }$ is the two-sided local neighborhood around $\theta_{l},$ as defined in (7) in [2]. Thus, the LMPU test is obtained by maximizing the power function, $\beta_{\Phi }\left(\theta_{l},\theta_{n}\right),$ among all locally unbiased tests under the α-size and unbiasedness constraints from (10) and (11) in [2], respectively, in the neighborhood of $\Omega_{\delta }$. The constraints from (10) and (11) in [2] can be rewritten by using (3.2) as follows:

(3.4) $$P_{FA}\left(\theta_{0},\theta_{n}\right)=\beta_{\Phi }\left(\theta_{l},\theta_{n}\right){|}_{\theta_{l}=\theta_{0}}=\alpha ,$$

(3.5) $$P_{D}\left(\theta_{l},\theta_{n}\right)=\beta_{\Phi }\left(\theta_{l},\theta_{n}\right){|}_{\theta_{l}\neq \theta_{0}}\geq \alpha ,\;\forall \theta_{l}\in \Omega_{\delta }.$$

Together, these constraints indicate that $\beta_{\Phi }\left(\theta_{l},\theta_{n}\right)$ has a minimum point at $\theta_{l}=\theta_{0}$ on the set $\Omega_{\delta }$. Since we assume that the common pdf, $f(x;\theta_{l},\theta_{n}),$ is twice differentiable in the local neighborhood of $\theta_{l}$ for any $\theta_{n}\in C^{K},$ the constraint in (3.5) can be replaced by the stationary condition

(3.6) $${\frac{\partial \beta_{\Phi }\left(\theta_{l},\theta_{n}\right)}{\partial \theta_{l}}|}_{\theta_{l}=\theta_{0}}=0,$$

together with the condition

(3.7) $${\frac{\partial^{2}\beta_{\Phi }\left(\theta_{l},\theta_{n}\right)}{\partial \theta_{l}^{2}}|}_{\theta_{l}=\theta_{0}}>0.$$

Therefore, by concluding the constraints in (3.4), (3.6), and (3.7), the LMPU test can be obtained from the solution of the following constrained optimization problem:

(3.8) $$\begin{array}{c} \max_{\Phi (x)}\beta_{\Phi }\left(\theta_{l},\theta_{n}\right)\;s.t.\;\left\{ \begin{array}{c} \beta_{\Phi }\left(\theta_{0},\theta_{n}\right)=\alpha \\ {\frac{\partial \beta_{\Phi }\left(\theta_{l},\theta_{n}\right)}{\partial \theta_{l}}|}_{\theta_{l}=\theta_{0}}=0\\ {\frac{\partial^{2}\beta_{\Phi }\left(\theta_{l},\theta_{n}\right)}{\partial \theta_{l}^{2}}|}_{\theta_{l}=\theta_{0}}>0,\;\forall \theta_{l}\in \Omega_{\delta } \end{array}\right.. \end{array}$$

Under the assumptions of differentiability, the Taylor series expansion of the power function, $\beta_{\Phi }\left(\theta_{l},\theta_{n}\right),$ around $\theta_{l}=\theta_{0}$ is given by:

(3.9) $$\begin{array}{cc} \beta_{\Phi }\left(\theta_{l},\theta_{n}\right) & =\beta_{\Phi }\left(\theta_{0},\theta_{n}\right)+\left(\theta_{l}-\theta_{0}\right){\frac{\partial \beta_{\Phi }\left(\theta_{l},\theta_{n}\right)}{\partial \theta_{l}}|}_{\theta_{l}=\theta_{0}}\\ & \;+\frac{1}{2}{\left(\theta_{l}-\theta_{0}\right)}^{2}{\frac{\partial^{2}\beta_{\Phi }\left(\theta_{l},\theta_{n}\right)}{\partial \theta_{l}^{2}}|}_{\theta_{l}=\theta_{0}}+O\left(\delta^{2}\right)\\ & =\alpha +{\left(\theta_{l}-\theta_{0}\right)}^{2}{\frac{\partial^{2}\beta_{\Phi }\left(\theta_{l},\theta_{n}\right)}{\partial \theta_{l}^{2}}|}_{\theta_{l}=\theta_{0}}+O\left(\delta^{2}\right), \end{array}$$

where the last equality is obtained by substituting the constraint on the false alarm probability from (3.4) and the unbiasedness constraint from (3.6). Thus, according to (3.9), in order to obtain the highest power, $\beta_{\Phi }\left(\theta_{l},\theta_{n}\right),$ for a given α and $\theta_{n},$ we need to maximize the second order term, ${\frac{\partial^{2}\beta_{\Phi }\left(\theta_{l},\theta_{n}\right)}{\partial \theta_{l}^{2}}|}_{\theta_{l}=\theta_{0}},$ for both $\theta_{l}>\theta_{0}$ and $\theta_{l}<\theta_{0},$ and this leads to the LMPU test. In addition, under the constraint $\beta_{\Phi }\left(\theta_{0},\theta_{n}\right)=\alpha ,$ (3.9) implies that the constraint in (3.7) is redundant for the maximization of $\beta_{\Phi }\left(\theta_{l},\theta_{n}\right)$ (which is always equal to or larger than α). Thus, the maximization in (3.8) is equivalent to the following optimization:

(3.10) $$\begin{array}{c} \max_{\Phi (x)}{\frac{\partial^{2}\beta_{\Phi }\left(\theta_{l},\theta_{n}\right)}{\partial \theta_{l}^{2}}|}_{\theta_{l}=\theta_{0}}\;s.t.\;\left\{ \begin{array}{c} \beta_{\Phi }\left(\theta_{0},\theta_{n}\right)=\alpha \\ {\frac{\partial \beta_{\Phi }\left(\theta_{l},\theta_{n}\right)}{\partial \theta_{l}}|}_{\theta_{l}=\theta_{0}}=0 \end{array}\right.. \end{array}$$

By using (3.2), it can be verified that

(3.11) $${\frac{\partial \beta_{\Phi }\left(\theta_{l},\theta_{n}\right)}{\partial \theta_{l}}|}_{\theta_{l}=\theta_{0}}={\frac{\partial }{\partial \theta_{l}}\left(\int_{\Omega_{x}}\Phi \left(x\right)f\left(x;\theta_{l},\theta_{n}\right)dx\right)|}_{\theta_{l}=\theta_{0}}.$$

Under the assumption that the test, Φ(x), is independent of the parameter $\theta_{l},$ the integration and derivatives in (3.11) can be reordered to obtain

(3.12) $${\frac{\partial \beta_{\Phi }\left(\theta_{l},\theta_{n}\right)}{\partial \theta_{l}}|}_{\theta_{l}=\theta_{0}}=\int_{\Omega_{x}}\Phi \left(x\right)\left(\frac{\partial f(x;\theta_{l},\theta_{n})}{\partial \theta_{l}}{|}_{\theta_{l}=\theta_{0}}\right)dx.$$

Similarly,

(3.13) $${\frac{\partial^{2}\beta_{\Phi }\left(\theta_{l},\theta_{n}\right)}{\partial \theta_{l}^{2}}|}_{\theta_{l}=\theta_{0}}=\int_{\Omega_{x}}\Phi \left(x\right)\left(\frac{\partial^{2}f\left(x;\theta_{l},\theta_{n}\right)}{\partial \theta_{l}^{2}}{|}_{\theta_{l}=\theta_{0}}\right)dx.$$

Therefore, by substituting (3.12) and (3.13) in (3.10), the integral form of (3.10) is

(3.14) $$\begin{array}{cc} & \max_{\Phi (x)}\int_{\Omega_{x}}\Phi \left(x\right)\left(\frac{\partial^{2}f\left(x;\theta_{l},\theta_{n}\right)}{\partial \theta_{l}^{2}}{|}_{\theta_{l}=\theta_{0}}\right)dx\\ s.t.\; & \left\{ \begin{array}{c} \int_{\Omega_{x}}\Phi \left(x\right)f\left(x;\theta_{0},\theta_{n}\right)dx=\alpha \\ \int_{\Omega_{x}}\Phi \left(x\right)\left(\frac{\partial f(x;\theta_{l},\theta_{n})}{\partial \theta_{l}}{|}_{\theta_{l}=\theta_{0}}\right)dx=0 \end{array}\right.. \end{array}$$

By using the auxiliary lemma of the Generalized Neyman-Pearson lemma (p. 77 in [1]) with m=2, $f_{1}=f\left(x;\theta_{0},\theta_{n}\right),$ $f_{2}={\frac{\partial f(x;\theta_{l},\theta_{n})}{\partial \theta_{l}}|}_{\theta_{l}=\theta_{0}},$ $f_{3}={\frac{\partial^{2}f\left(x;\theta_{l},\theta_{n}\right)}{\partial \theta_{l}^{2}}|}_{\theta_{l}=\theta_{0}},$ $c_{1}=\alpha ,$ and $c_{2}=0,$ the LMPU test that solved (3.14) rejects the null hypothesis when

(3.15) $${\frac{\partial^{2}f\left(x;\theta_{l},\theta_{n}\right)}{\partial \theta_{l}^{2}}|}_{\theta_{l}=\theta_{0}}>{\tilde{\kappa }}_{1}{\frac{\partial f(x;\theta_{l},\theta_{n})}{\partial \theta_{l}}|}_{\theta_{l}=\theta_{0}}+{\tilde{\kappa }}_{2}f\left(x;\theta_{0},\theta_{n}\right),$$

where the constants ${\tilde{\kappa }}_{1}$ and ${\tilde{\kappa }}_{1}$ are chosen such that the constraints in (3.14) (or, equivalently, the constraints in (3.10)) are satisfied. That is, ${\tilde{\kappa }}_{1}$ and ${\tilde{\kappa }}_{1}$ are chosen such that $\beta_{\Phi }\left(\theta_{0},\theta_{n}\right)=\alpha$ and ${\frac{\partial \beta_{\Phi }\left(\theta_{l},\theta_{n}\right)}{\partial \theta_{l}}|}_{\theta_{l}=\theta_{0}}=0$ are satisfied for the test Φ defined by (3.15). In addition, it can be verified that

(3.16) $${\frac{\partial f(x;\theta_{l},\theta_{n})}{\partial \theta_{l}}|}_{\theta_{l}=\theta_{0}}={\frac{\partial \log f(x;\theta_{l},\theta_{n})}{\partial \theta_{l}}|}_{\theta_{l}=\theta_{0}}f\left(x;\theta_{l},\theta_{n}\right){|}_{\theta_{l}=\theta_{0}}$$

and

(3.17) $$\begin{array}{c} {\frac{\partial^{2}f\left(x;\theta_{l},\theta_{n}\right)}{\partial \theta_{l}^{2}}|}_{\theta_{l}=\theta_{0}}\;\\ =\left(\frac{\partial^{2}\log f\left(x;\theta_{l},\theta_{n}\right)}{\partial \theta_{l}^{2}}\right){|}_{\theta_{l}=\theta_{0}}f\left(x;\theta_{l},\theta_{n}\right){|}_{\theta_{l}=\theta_{0}}+\frac{\partial f(x;\theta_{l},\theta_{n})}{\partial \theta_{l}}{{|}_{\theta_{l}=\theta_{0}}\frac{\partial \log f(x;\theta_{l},\theta_{n})}{\partial \theta_{l}}|}_{\theta_{l}=\theta_{0}}. \end{array}$$

By substituting (3.16) into (3.17), one obtains

(3.18) $$\begin{array}{c} \frac{\partial^{2}f\left(x;\theta_{l},\theta_{n}\right)}{\partial \theta_{l}^{2}}{|}_{\theta_{l}=\theta_{0}}\;\\ =\left(\frac{\partial^{2}\log f\left(x;\theta_{l},\theta_{n}\right)}{\partial \theta_{l}^{2}}\right){|}_{\theta_{l}=\theta_{0}}f\left(x;\theta_{l},\theta_{n}\right){{|}_{\theta_{l}=\theta_{0}}+{\left(\frac{\partial \log f(x;\theta_{l},\theta_{n})}{\partial \theta_{l}}{|}_{\theta_{l}=\theta_{0}}\right)}^{2}f\left(x;\theta_{l},\theta_{n}\right)|}_{\theta_{l}=\theta_{0}}. \end{array}$$

Then, by substituting (3.16) and (3.18) in (3.15), we get that the LMPU test that solved (3.14) is the LMPU test for a known parameter vector, $\theta_{n},$ given in (12) in [2].

## 4 Code Files

The source code for the LMPU and GLMPU functions for the special case of testing frequency deviations is given below. The MATLAB scripts are used to generate the simulations in [2], as follows:

• LMPU_FUN.m - calculates the LMPU test from (21) in [2] for the detection of a small deviation in the frequency of sinusoidal signals, where the amplitudes and noise variance are unknown.
• GLMPU_FUN.m- calculates the GLMPU test from (23) in [2] for the detection of a small deviation in the frequency of sinusoidal signals, where the amplitudes and noise variance are unknown.

## Ethics Statement

The authors declare that this work has been done according to the ethical requirements for publication in Data in Brief.

## Declaration of Competing Interest

The authors declare that they have no known competing financial interests or personal relationships which have, or could be perceived to have, influenced the work reported in this article.
